# Lesson learned from assessing teachers’ and students’ perspectives regarding the quality of e-learning in medical education during the pandemic: a mixed-methods study

**DOI:** 10.1186/s12909-024-05160-4

**Published:** 2024-02-22

**Authors:** Nahid Zarifsanaiey, Majid Reza Farrokhi, Zahra karimian, Sara hoseini, farshid chahartangi, Hadi Raeisi Shahraki

**Affiliations:** 1 Department of E-learning, Virtual School, Shiraz University of Medical Sciences, Shiraz, Iran; 2 Department of Neurosurgery, School of Medicine, Neuroscience Research Centre, Shiraz University of Medical Sciences, Shiraz, Iran; 3 Shiraz University of Medical Sciences, Shiraz, Iran; 4 Departments of Epidemiology and Biostatistics, Faculty of Health, Shahrekord University of Medical Sciences, Shahrekord, Iran

**Keywords:** Quality, E-learning, Evaluation, Medical University, Students, Perspective

## Abstract

**Background:**

The evaluation of e-learning systems ensures the provision of quality training. The goal was to identify the perspectives of teachers and students on e-learning in medical education during the COVID-19 pandemic at Shiraz University of Medical Sciences (SUMS), Iran.

**Methods:**

This study utilized a convergent mixed methods research design with a two-phase approach to collect and analyze data between June and August 2022. In the first stage, a cross-sectional descriptive study was conducted to evaluate the quality of e-learning systems from the perspective of 400 students. In the second stage, semi-structured interviews were conducted with 10 virtual education professors and 10 student representatives to identify the strengths, weaknesses, opportunities, and threats of virtual education. A validated questionnaire was administered to assess the quality of the e-learning system, and data were analyzed using SPSS-21. Qualitative data were subjected to content analysis.

**Results:**

Our findings revealed that the student support system, the course structure, and the infrastructure and technology subscales’ mean scores were significantly higher than the average level (*P* < 0.001). However, the professors’ methods of teaching and learning strategies were unsatisfactory. The results of the present study showed that the evaluation mean score was significantly higher among, younger, female, and undergraduate students. Virtual education has strengths and weaknesses, and innovative approaches are needed to enhance student engagement. The lack of appropriate infrastructure and virtual teaching tools for teachers and students is a significant challenge that needs to be addressed. Blended learning is effective in medical education, and the shift from teacher-centered to learner-centered teaching approaches is an opportunity to explore innovative teaching approaches.

**Conclusion:**

From the perspective of students, the quality of eLearning systems at the universities was moderate. Virtual education offers both benefits and drawbacks, and there is a requirement for innovative solutions to enhance student engagement and lessen boredom.

## Background

In the era of e-learning with COVID-19 impacting the latter months of 2019, the education system had to adapt to new ways of teaching and learning [1].

The medical education sector has experienced a significant shift towards e-learning as the pandemic forced students to learn remotely. Consequently, medical universities rapidly transitioned to e-learning to ensure continuity of education [2]. This complex process involved both teachers and students in the implementation of e-learning [3]. E-learning, which involves the use of digital technologies and the internet to deliver educational content, has revolutionized teaching and interaction techniques, enabling education to continue regardless of time constraints. It encompasses accessing materials, participating in interactive lessons, and collaborating with instructors and peers through computers, mobile devices, and online platforms. E-learning offers flexible learning options in terms of time, location, and pace, enabling individuals to access resources and engage in activities at their convenience. Universities and educational institutions have been able to provide services to students more flexibly, ensuring connectivity and the ability to continue studies even during crisis situations [4].

The success of any educational system depends on the participation of its students and teachers [5]. Therefore, their opinions are considered to be one of the most crucial factors in the success of e-learning systems [6]. Numerous studies have been administered in an effort to gauge teachers and students’ perspectives toward e-learning. For instance, a study conducted by Dyrek et al. in Poland showed that students of Medical Universities were highly accepting of lectures and seminars conducted through e-learning, but not laboratory and clinical classes [7]. On the other hand, Rathod et al. highlighted the flexibility and convenience provided by e-learning systems, which allow students to access educational content at their own pace and time [8]. Moreover, e-learning could be cost-effective in the long run and address the problem of travel distance and time [9]. In this regard, a study by Li et al. found that time, place, learning speed, and flexibility are critical factors in students’ decisions to use the e-learning system [10]. In addition, e-learning has ensured the continuity of education during the COVID-19 pandemic, allowing students to continue their studies despite the challenges posed by the pandemic [11].

Despite the numerous benefits of e-learning, designing a functional e-learning system remains a significant hurdle. The major hurdles that undermine student and teacher satisfaction include a lack of suitable infrastructure, inadequate content development, insufficient teacher-student interaction, ineffective online teaching methods, as well as restricted access to technology [12–14].. Furthermore, In some studies about the challenges of e-learning during the COVID-19 pandemic specially in low- and middle-income countries showed that students and faculty may find it difficult to use e-learning systems effectively due to resource constraints, technological barriers, and cultural differences [15].

In response to the COVID-19 pandemic and its impact on the teaching-learning process, Shiraz University of Medical Sciences (SUMS) conducted a sudden switch to online learning, disrupting traditional practices of instruction and assessment. While the university had made efforts to introduce e-learning workshops over the last decade, many teachers and students were not well-versed with online teaching before the pandemic. This move also raised questions about the quality of teaching and learning. To address the issue, the university conducted additional workshops to train students and teachers to work with the university’s Learning Management System (LMS), virtual classes, discussion forums, and assigned names and passwords to all students and teachers to work with the platforms. Therefore, they used LMS, virtual classes, discussion forums, and other open-access social media, such as Zoom and Skype, to design their courses synchronously and asynchronously. In addition, the university provided a technical support system that was available seven days a week for teachers and students to address technical issues during online learning sessions. Due to its significant impact on the education system, COVID-19 has accelerated the implementation of e-learning worldwide, making it crucial to evaluate its effectiveness [16]. To ensure the effectiveness of e-learning in medical education, it is crucial to evaluate the perceptions of students and teachers. Considering that the evaluation of students’ and teachers’ perceptions of the educational system plays a significant role in enhancing educational quality and that there is a perceived information gap in e-learning in medical universities [16–17]. As medical education is undergoing a transformation worldwide, it is crucial for universities and planners to critically reflect on the previous situation and make appropriate decisions regarding the future of medical education because e-learning has specific benefits and challenges in each university and it is very important to analyses. Most related research focuses on one of the crucial components of the education system, such as teachers and students, and examines their perspectives [14–15]. In order to gain a more comprehensive understanding of the views of both instructors and students, we conducted a mixed-method study that combined qualitative and quantitative research.” Therefore, this study aimed to evaluate e-learning quality from both the perspectives of teachers and students during the COVID-19 pandemic. Lessons learned and feedback obtained from the evaluation process can greatly impact the design and implementation of future e-learning initiatives, ensuring a high-quality learning experience for students.

## Methods

### Study design

This study employed a convergent mixed methods research design with a two-phase approach to collect and analyze data. The first stage involved a cross-sectional descriptive study to evaluate the quality of e-learning systems from the perspective of students affiliated with SUMS.

Subsequently, in the second phase, semi-structured interviews were conducted with two groups: ten responsible virtual education professors and ten student representatives. The purpose of these interviews was to gain deeper insights into the experiences and perspectives of both teachers and students regarding e-learning in the medical education field. By utilizing semi-structured interviews, the researchers aimed to identify specific strengths, weaknesses, opportunities, and threats associated with virtual education.

By combining the findings from both the cross-sectional study and the interviews, the researchers aimed to generate valuable insights into the overall e-learning quality from both the perspectives of teachers and students during the COVID-19 pandemic.

### Participants

Eligibility criteria for participants:

In the quantitative stage, the study included undergraduates and graduate medical students at SUM. These students had completed at least two semesters of virtual classes using synchronous and asynchronous methods. The virtual classes were conducted through the university’s Learning Management System (LMS), along with discussion forums and other open-access platforms like social media, Zoom, and Skype. These students met the inclusion criteria for the study. It is worth noting that all the universities in the region use the same learning management system (LMS) for educational activities. They completed informed consent forms and were willing to participate. Those who did not complete more than 20% of the questionnaires were excluded from the study. In total, about 10,000 people were eligible to participate in the study.

In the qualitative stage, ten representatives of students, and 10 professors who have been responsible for developing virtual education in colleges for at least 6 months were selected. Participants who did not complete the interview process were excluded from the study. Both individuals were selected based on the expectation that they would possess the ability to provide thorough and detailed information pertaining to the research goal.

### Sampling

The quantitate stage: To determine the sample size, Cochran’s formula and Morgan’s table were used. Taking into account a 95% confidence interval and a 20% attrition rate, 400 participants were considered.

$$n\,{\text{=}}\,\frac{{\frac{{{z^2}pq}}{{{d^{\text{2}}}}}}}{{{\text{1}}\,{\text{+}}\,\frac{{\text{1}}}{N}\left[ {\frac{{{z^2}pq}}{{{d^2}}}\, - \,{\text{1}}} \right]}}$$

The research samples were selected by convenience sampling method during June to august 2022 [18]. Sampling continued until the number of completed questionnaires reached 400.

The qualitative stage: At this stage, representatives of students and experienced professors in the field of virtual education were purposefully selected until data saturation was achieved.

### Data collection tools

The data collection tool consisted of two sections, the first of which dealt with demographic details (age, gender, GPA (grade point average), grade, year of study, and faculty). The second section allocated with students’ opinions on the quality of e-learning systems, measured on a Likert scale from 1 (strongly disagree) to 5 (strongly agree) This section has 27 items in 5 domains: teaching-learning strategies (design, evaluation, and feedback) (9 items), course structure (consistency of the curriculum, compliance with laws and ethical standards, easy access to the education space) (9 items), infrastructure and technology (necessary software, network speed, the ability to set up audio and video, the quality of multimedia files, and the security of students’ information) (10 items). Test- value is this study is 2.50.

The Questionnaire was developed by Shahhoseini et al. in 2015 as a standardized tool to measure the quality of e-Learning systems. The questionnaire has a high content validity index (CVI) of 0.94, with a content validity ratio (CVR) of 0.87. The questionnaire’s reliability has been found to be acceptable, with a reliability of 94.5% [19]. In addition, the Pearson correlation test in this study showed that there is a positive and significant correlation between the total score of the questionnaire and each sub-questionnaire (*P* < 0.001) (Table 1). The high Pearson correlation coefficients between the subscales of the questionnaire (ranging from 0.68 to 0.92) suggest a strong positive relationship between the dimensions, indicating good internal consistency and reliability of the questionnaire. This is supported by the concept that reliability ensures consistency in the results, and the high correlations reflect the consistent relationships between the subscales over time and across similar conditions [20].

**Table 1:** The Pearson correlation between the subscales of the questionnaire

Dimension	Teaching-learning strategies	student Support system	Course structure	Infrastructure and technology
Total	0.88	0.90	0.92	0.92
Teaching-learning strategies	1	0.68	0.72	0.75
student Support system		1	0.83	0.78
Course structure			1	0.92
Infrastructure and technology				1

All the correlations were significant (*P* < 0.001)

Initially, eligible students were provided online questionnaires 24 h after completing the informed consent form. Those who did not return questionnaires were contacted and urged to do so. Noticeably, only one follow-up effort was conducted per participant.

Qualitative data were collected through online interviews conducted on the WhatsApp social network. The aim of these interviews was to identify the strengths, weaknesses, opportunities, and threats associated with virtual education through SWOT analysis. SWOT analysis is a commonly used tool to identify the strengths, weaknesses, opportunities, and threats in an educational environment. It helps to identify the factors that can affect the success of virtual education and can be used to develop strategies to address these factors [21–22].

To further improve the validity and reliability of the study, several steps were taken. During the interviews, the discussions were audio-recorded to capture the participants’ responses accurately. These recordings were later transcribed verbatim, ensuring that no crucial details were missed during the analysis. By using verbatim transcripts, the researchers aimed to maintain the integrity and accuracy of the data [23].

Then, member checking was employed. After transcribing the interview discussions verbatim, the transcripts were shared with the 20 participants who took part in the semi-structured interviews. This process involved seeking feedback from the participants to ensure the accuracy and credibility of the data. The participants were given the opportunity to review their respective transcripts and provide input regarding the accuracy of the transcriptions and the interpretation of their responses. This step allowed them to confirm whether their viewpoints were adequately represented and if any necessary revisions or clarifications were required. In addition, the validity of the interview content was also confirmed by 5 experts in e-learning and medical education. These external experts reviewed the interview transcripts and provided their professional insights and expertise to validate the accuracy of the data interpretation. Their expertise and feedback added credibility and rigor to the study. In addition, the authors’ team conducted multiple meetings at each stage, fostering dynamic discussions, facilitating comparative interpretations, and ultimately reaching a consensus on data analysis.

The interview questions were as follows:

1. What are the strengths of virtual education in medical sciences?
2. What are the weaknesses of virtual education in medical sciences?
3. What opportunities exist to improve virtual education?
4. What threats are there to virtual education in medical sciences?

### Statistical methods

SPSS 21 was used to analyze the collected data. The data was then analyzed using descriptive statistics, as well as Pearson’s correlation coefficient, independent T-test and one way analysis of variance (ANOVA), to determine whether there was a relationship between the demographic data and opinions on the quality of e-learning systems.

The qualitative data were subjected to content analysis, with three authors manually analyzing the data. Through a meticulous process of coding and categorizing, themes emerged to encapsulate the essence of the data. The study also followed the Consolidated Criteria for Reporting Qualitative Research (COREQ) checklist to ensure transparency and rigor in the reporting of the study’s methods and results.

### Data integration

The quantitative and qualitative data were integrated by comparing and contrasting the findings. The qualitative data were used to explain and build upon the initial quantitative results.

### Ethical considerations

The study commenced following approval by the local ethics committee and coordination with the vice president of research at the universities. In the initial phone call, the researcher explained the objectives of the study, emailed the informed consent form to the participant, and obtained their signed informed consent. To ensure anonymity, no names were included on the surveys, and a research assistant decoded all completed questionnaires to prevent errors. To adhere to ethical standards, participation was voluntary and participants had the right to withdraw at any time.

## Results

The 400 recruited students were enrolled through convenience sampling. Most of the participants were male (56.8%), between 18 and 22 years (53.0%), bachelor’s degree (57.3%) with GPA 16 to 17.99 (52.5%). The mean and standard deviation of the evaluation score according to demographic variables can be seen in Table 2.

**Table 2:** The relationship between demographic characteristics and mean the evaluation score

Variable	Sub group	N (%)	Mean ± SD	P-value
Gender	Female	173 (43.3)	2.86 ± 0.52	< 0.001
	Male	227 (56.8)	2.62 ± 0.59	
Age	18–22 years	212 (53.0)	2.85 ± 0.55	< 0.001
	23–27 years	131 (32.8)	2.62 ± 0.58	
	28–32 years	44 (11.0)	2.51 ± 0.48	
	+ 32 years	13 (3.3)	2.37 ± 0.66	
GPA (Grade Point Average)	< 14	5 (1.3)	2.75 ± 0.53	0.52
	14-15.99	81 (20.3)	2.74 ± 0.57	
	16-17.99	210 (52.5)	2.68 ± 0.62	
	18–20	104 (26.0)	2.42 ± 0.35	
Grade	Bachelor	229 (57.3)	2.85 ± 0.55	< 0.001
	Master	20 (5.0)	2.58 ± 0.44	
	Professional doctor	102 (25.5)	2.54 ± 0.62	
	Ph. D	49 (12.3)	2.57 ± 0.48	
Year of study	First or second	88 (22.0)	2.76 ± 0.55	< 0.001
	3rd or 4th	120 (30.0)	2.85 ± 0.55	
	5th or 6th	105 (26.3)	2.74 ± 0.59	
	7th or 8th	53 (13.3)	2.56 ± 0.56	
	Higher	34 (8.5)	2.36 ± 0.50	
School	Medical	120 (30.0)	2.56 ± 0.58	< 0.001
	Dentistry & Pharmacy	40 (10)	2.38 ± 0.65	
	Nursing & Midwifery	89 (22.3)	3.04 ± 0.53	
	Management &|Health	60 (15.0)	2.81 ± 0.45	
	paramedical & Rehabilitation	91 (22.7)	2.78 ± 0.51	

There was a significant relationship between participants’ scores and age, gender, grade, year of study, and faculty (< 0.001). The female perspective mean score was significantly higher among male students. Furthermore, the mean score of the views decreased significantly with age. Also, undergraduate students compared to other courses and students of other universities had a better view of the state of the electronic learning system than students at Shiraz University of Medical Sciences. Nursing students with 3.04 ± 0.53 and dentistry students with 2.38 ± 0.65 respectively had the highest and lowest average score. Table 3 shows the mean score of the participants’ perspective subscales.

**Table 3:** The difference of evaluation score among the subgroup of demographic characteristics

Variable	Mean ± SD	Test value	Mean difference	T-value	P-value
Total	2.73 ± 0.57	2.50	+ 0.23	7.78	< 0.001
Teaching-learning strategies	2.47 ± 0.67	2.50	-0.03	0.92	0.36
student Support system	2.81 ± 0.56	2.50	+ 0.31	10.9	< 0.001
Course structure	2.90 ± 0.66	2.50	+ 0.40	12.12	< 0.001
Infrastructure and technology	2.74 ± 0.64	2.50	+ 0.24	7.57	< 0.001

The findings revealed several important aspects of e-learning in the medical education context. Our findings revealed that the student support system, the course structure, and the infrastructure and technology subscales’ mean scores were significantly higher than the average level However, the teaching-learning strategies subscale mean score was lower than the average level, indicating that there is room for improvement in this aspect of the educational environment -learning strategies were unsatisfactory. In addition, the total mean score of students was significantly higher than the average level (*P* < 0.001).

According to the views of 20 experts and students in this field, the strengths, weaknesses, threats, and opportunities of virtual education were categorized as follows:

### The student’s perspective

#### Strengths

One of the strengths identified by students was the flexibility of e-learning. They appreciated the ability to learn at their own pace and schedule, which allowed them to balance their academic pursuits with other commitments. The accessibility of educational materials and guidelines was also highlighted as a strength, as students could access resources from anywhere, eliminating the need for physical presence in a classroom.

It has many strengths as a form of education. One of the most notable advantages of e-learning is convenience. As students, we can access our classes and materials from anywhere, at any time. This flexibility allows us to work around our other commitments, and makes it easier to balance our academic and personal lives. Participating student No.2.

The most strength of e-learning is its ability to personalize the learning experience. Through quizzes and assessments, we can track our progress and identify areas where we need to improve. This can help us set goals and make progress towards them, which can be highly motivating. Additionally, the availability of online learning materials means that students have access to a wealth of information and resources, allowing us to learn and grow even beyond the classroom setting. Participating student No.4.

### Weaknesses

Based on the provided information, it is evident that the teaching approaches implemented in the e-learning environment were deemed unsuitable, resulting in online classes being perceived as tedious and uninteresting. This highlights a weakness in the execution of teaching methods within the virtual setting. The lack of student engagement was a prominent issue observed in the online classes. The strategies employed failed to effectively captivate students, leading to a noticeable absence of interest and a sense of boredom. The absence of face-to-face interactions with peers and instructors could deprive students of the social aspect of traditional classrooms. This reduced social interaction may impact their motivation, sense of belonging, and ability to form meaningful relationships with fellow students and teachers.

The repetitive use of similar instructional methods without variation further compounded the students’ perception of boredom. Additionally, the insufficient utilization of interactive tools, such as virtual simulations, multimedia presentations, or interactive quizzes, hindered active participation and engagement, exacerbating the lack of student involvement. These factors collectively underscore the weaknesses of the teaching approaches in terms of insufficient interaction, inadequate variation of instructional methods, and limited use of interactive tools.

While e-learning has many benefits, it also has its weaknesses. One of the main problems is the lack of personal interaction with the teacher and other classmates. This can make it difficult to ask questions and get feedback, which can hinder understanding and learning. Additionally, the need for self-discipline and motivation can be a challenge for some students. Some students may struggle to stay focused and manage their time when learning online, which can impact their performance. Furthermore, e-learning can be limited in terms of the practical and hands-on experience that students need to develop certain skills. This can make it difficult to apply theoretical knowledge in a practical setting. Participating student No.3.

### Opportunities

The given information suggests that there are weaknesses in the current teaching and learning strategies applied in e-learning environments. However, these weaknesses present an opportunity to explore and implement innovative approaches that are better suited for virtual education. To enhance student engagement and reduce boredom associated with traditional lecture-based approaches, active learning strategies such as, case studies, group discussions, and virtual collaborative projects can be implemented. Additionally, effective use of technology, including interactive online platforms, virtual reality simulations, and gamification elements, can create more dynamic and engaging learning experiences for students.

E-learning has opened up a world of opportunities for me. I can access course materials and lectures at any time, from anywhere, which has been incredibly convenient. It has also allowed me to explore a wider range of subjects and learn from professors and experts around the globe, which I may not have had access to otherwise. Participating student No. 8

### Threats

Without proper infrastructure and the use of innovative technology such as simulation, gamification, and effective instruction, classes may become boring, and students may struggle to complete learning activities on time. It is crucial to create motivation, interest, and establish communication with students, and ignoring these issues can lead to fundamental problems.

E-learning has been a mixed experience for me. While it has allowed me to access course materials and lectures at any time, from anywhere, it has also been difficult to stay motivated and engaged without the structure of in-person classes. Additionally, technical issues and poor internet connectivity have been a major source of frustration.

### The professor’s insight

#### The strengths

Virtual education has several strengths that have been identified by experts. One of the main strengths of virtual education is flexibility and easy access to learning materials anytime and anywhere, which allows learners to practice and repeat their learning activities at their own pace until they reach mastery. However, they also emphasized that online assessment methods should be compatible with the learner-centered approach. They emphasized the use of blended learning in medical education and believed that it is one of the most effective ways that can replace purely virtual courses. According to them, virtual education optimizes the use of human resources and prevents occupational burnout among instructors and staff. Moreover, students with different conditions and occupations such as employment and marital status can benefit from this approach. They stressed that virtual courses, if well-designed and attention is paid to visual, audio, and other content aspects that are relevant to the content, can also help to enhance the depth and durability of learning. On the other hand, they mentioned that virtual education can provide more calmness and motivation for instructors alongside face-to-face teaching, given the heavy and multi-faceted tasks of faculty members.

In fact, we are able to use technological advancements, such as LMS and online platforms, in education to facilitate teaching and learning. This allows individuals to learn remotely, which can be more efficient and time-saving. It also allows students to take on more responsibility for their learning and be more accountable for their progress. However, effective assessment methods are necessary to evaluate the knowledge that has been conveyed to the student. Participating teacher No.3.

Utilizing and optimizing the benefits of remote learning can provide a more efficient and effective learning experience for students and faculty. This includes making education accessible to students who may have difficulty attending in-person classes, such as those who are married, employed, or otherwise unable to attend class. Well-designed online courses that utilize effective visuals, audio, and other techniques can enhance the depth and retention of learning. Additionally, offering remote learning options can reduce the burden and stress on members of the faculty, which can lead to a more motivated and engaged teaching experience. Participating teacher No.7.

#### Weaknesses

Experts agree that virtual education has many strengths, but it also has weaknesses, particularly in the field of medical education and learning. One of the drawbacks is that teachers may not be familiar with the philosophy, teaching strategies, and production of e-content, leading to low-quality courses. Additionally, the lack of appropriate infrastructure and virtual teaching tools for teachers and students, as well as their unfamiliarity with the hardware and software required for teaching and producing suitable content, can hinder effective learning, especially during the COVID-19 era.

Furthermore, experts also noted that there is a lack of motivation among teachers to produce creative content and design innovative courses, resulting in delivering uniform and tedious education. They also identified the inability to use state-of-the-art technologies, particularly in clinical education, as a significant challenge. Experts agree that virtual education is not suitable for practical courses, apprenticeships, and bedside teaching, as learners cannot experience the real environmental challenges.

Another weakness of virtual education is the lack of face-to-face and close communication between teachers and students, which can negatively impact relationship-building, motivation, and effective teaching. The absence of students in the university environment and the disregard for the hidden curriculum can also affect the level of learning and students’ role as active and effective learners. Virtual education alone cannot provide the social and academic growth that comes with direct relationships between classmates and teachers, leading to isolation. Lastly, experts highlighted the inability to actively monitor students’ activities, especially in virtual classes, and provide accurate assignments, which can hinder effective learning. Online learning environments may have more distractions compared to traditional classroom settings, making it harder for students to stay focused and attentive. Furthermore, e-learning demands self-motivation and discipline from students. Without the physical presence of teachers and the structure of a traditional classroom, students must rely on their own self-discipline to stay engaged, manage their time effectively, and complete their coursework. This requires a higher level of self-motivation and can be challenging for some students.

The lack of interaction in online learning is one of the biggest challenges. For example, if the course is designed as pre-recorded lectures without a virtual classroom, there’s no feedback or interaction with the students. Some courses cannot be taught effectively in an online format, such as courses with a heavy emphasis on practical work or hands-on experiences. Additionally, interaction between professors and students is crucial for effective learning. Online learning can limit this interaction, and it’s difficult to understand students’ needs and provide feedback without it. Participating teacher No.1.

When students have access to the internet during virtual classes, they may not pay close attention or use outside resources instead of focusing on the class material. Virtual exams are challenging to design and assess effectively. They may not accurately reflect students’ knowledge and abilities. The transition to online learning was rushed, and instructors were not fully prepared. This led to chaos and inefficiency in the system. Monitoring and reporting requirements in online learning often impose unnecessary workload on instructors, taking away time and attention from teaching. Online learning platforms can sometimes experience technical disruptions or connectivity issues. This can cause problems for students and instructors. Overall, the biggest weakness with online learning is the lack of interaction and engagement between professors and students. This can lead to a lower quality of education and a less effective learning experience. It’s important to consider the benefits and drawbacks of online learning before implementing it. Participating teacher No.5.

#### Opportunities

According to experts, there are many opportunities created by the appropriate combination of education and technology. By making proper use of modern technologies, especially various simulation methods, the gap between theory and practice can be reduced. Moreover, these methods can strengthen students’ procedural and clinical decision-making skills, prepare them to face real clinical challenges, and reduce medical errors and stress. Currently, students immediately enter the clinic after theory, which carries a lot of human error and stress. These methods can also be used effectively in community health and patient education. If virtual courses are designed in accordance with current global standards and all the capabilities of the learning management system are utilized, education will become more flexible, and many of the cumbersome processes will be reduced because all the activities of the instructor and student are recorded and traceable. Based on this, more accurate evaluation can be done.

According to professors, the most important opportunity before the rapid change of the educational system to in-person learning is the presence of trained human resources in universities. After two years, professors and experts have become familiar with the necessity, utility, and implementation of some systems, which is a good opportunity to improve and overcome weaknesses in in-person education. Also, given that most students belong to the digital age, virtual education provides an opportunity for teachers and universities to adapt to the needs of their students and provide more attractive and flexible education. Blended learning can provide more cost-effective opportunities for universities in today’s conditions by reducing many expenses such as transportation, nutrition, etc.

If the right conditions are in place, it would be beneficial for medical students to use technology such as virtual reality and simulation for clinical training. It could help them learn about medical equipment and procedures in a more interactive and engaging way, and potentially reduce the reliance on traditional methods. By incorporating technology into their education, medical students can gain valuable experience with modern medical practices, and be better prepared for their careers. Additionally, this could help reduce the workload for instructors and enhance the learning experience for students. However, it’s important to provide adequate training and resources to ensure that the technology is used effectively and safely. Participating teacher No.5.

Opportunity to leverage the trained human resources in Universities is a crucial factor in the current scenario, as it provides a chance to improve and address the shortcomings in the use of online learning platforms. Professors and experts have also gained experience in the use of these systems, providing an opportunity to integrate their knowledge into the educational system. This can help enhance the quality of education and ensure that students are equipped with the necessary skills for the future. It is crucial to leverage such opportunities and continuously explore ways to improve the effectiveness of online learning platforms. This can help address the challenges faced by students and instructors in the current situation, and ensure the continuity of education in a more productive and effective way. Participating teacher No.1.

#### Threats

According to experts, if there is no proper teaching- learning strategy and monitoring, students may not attend classes and not perform learning activities on time. Motivation, interest, and establishing a connection with students may be challenging in a virtual environment. In addition, the quality of virtual education may be lower than in-person education if the necessary infrastructure, technology, and support are not provided. Therefore, proper planning, design, implementation, and monitoring are essential for virtual education to be effective. Virtual education may lead to feelings of isolation and reduced social interaction among students, impacting their overall well-being and sense of belonging. Ensuring consistent quality in virtual education can be challenging, as it requires effective monitoring and evaluation to maintain standards and ensure the delivery of high-quality content. Assessing student learning and providing authentic feedback in virtual education can be more challenging than in traditional classroom settings, as it may require alternative assessment methods and tools.

The lack of attention to the problems and concerns raised by users is highlighted as a serious threat to the long-term success of the online learning system. This negligence can result in weaknesses in the system and a decline in its effectiveness over time. Several factors contribute to this threat, including inadequate infrastructure, the lengthy process of implementing new online learning methods, and the cost of certain techniques such as simulation and virtual reality. Additionally, the lack of consideration of the positive aspects of each program, the rapid shift from one method to another, and the absence of close communication between users and administrators are identified as potential threats. Participating teacher No.10

### Combined insights

By combining the findings from the cross-sectional study and the interviews, the researchers generated valuable insights into the overall effectiveness and impact of virtual education in the medical field. The strengths, weaknesses, opportunities, and threats identified provided a comprehensive understanding of the challenges and benefits of e-learning in medical education during the COVID-19 pandemic.

Based on the provided information, the strengths, weaknesses, opportunities, and threats of virtual education can be identified in relation to teaching-learning strategies, course structure, and infrastructure and technology domains in Table 4.

**Table 4:** Category, Sub-category, and themes

Category	Sub-category	Them
Teaching-Learning Strategies	Strengths	Flexibility, learning at their own pace and convenience, access to resources, self-paced learning
	Weaknesses	Inadequate teaching approach, insufficient interaction, hindrance of immediate feedback and personal connections, challenges in effectively delivering clinical education activities, inadequate variation of instructional methods, limited use of interactive tools, potential impact on motivation and engagement
	Opportunities	Exploring innovative teaching approaches, incorporating active learning strategies, leveraging technology, blended learning, address different learning styles
	Threats	Independent nature of virtual learning requiring self-motivation, time management skills, and self-discipline from students
Course Structure	Strengths	Consistency and compliance, well-structured virtual courses, following a consistent curriculum, ensuring compliance with ethical standards, and educational guidelines, providing students with a structured learning experience, easy access, online platforms allowing students to navigate the course content conveniently at their own pace
	Weaknesses	absence of in-person guidance and supervision in virtual education, potential gaps in understanding or lack of clarity in course content, need for students to be more proactive in seeking clarifications, limited Social Interaction’ lack of social interactions in virtual education, potential impact on the development of interpersonal skills and peer support networks
	Opportunities	Accessibility to education for individuals facing geographical or time constraints, enabling lifelong learning and reaching a broader audience who may not have had access to traditional education, flexibility of online learning allowing for seamless integration of education with work commitments, virtual education allowing students from diverse backgrounds and regions to interact, fostering cultural exchange and global collaboration, expanding students’ perspectives through exposure to different viewpoints and experiences, unique and vital traits of online learning format
	Threats	Unequal access to technology and reliable internet connections can create disparities in educational opportunities, hindering some students’ ability to fully participate in virtual education and exacerbating existing educational inequalities, ensuring consistent quality across virtual courses and assessing learning outcomes effectively can be challenging in the absence of traditional evaluation methods, requiring robust mechanisms for quality assurance and accreditation.
Infrastructure and Technology	Strengths	Availability of necessary software, the availability of appropriate software and tools, facilitating seamless online learning experiences, learning management systems, video conferencing platforms, and interactive learning tools, easy setup of audio and video, ability to set up audio and video connections in virtual education, enabling real-time interactions between students and instructors, facilitating live lectures and discussions
	Weaknesses	Technical challenges, software compatibility problems, unreliable internet connections, limited access to necessary devices,
	Opportunities	Opportunities for innovative technology virtual education, leveraging digital tools and multimedia content to enhance the learning experience, incorporating interactive elements such as videos, quizzes, and simulations, making the learning process more engaging and effective, potential for global collaboration and cultural exchange among students from different regions broadening perspectives and promoting intercultural understanding
	Threats	Digital divide and unequal access to technology, threats to the overall effectiveness of virtual education, Lack of access to necessary devices or reliable internet connections, creating educational inequalities and hindering students’ ability to fully participate in e-learning, social isolation and effective assessment and feedback, potential for social isolation in virtual education, need for effective assessment and feedback in the e-learning environment, absence of face-to-face interactions and challenges of providing timely and meaningful feedback in virtual settings
Support system	Strengths	Accessibility, flexibility, variety of support options, collaboration opportunities, personalized support
	Weaknesses	Lack of face-to-face interaction, technical difficulties, limited non-verbal cues, potential for social isolation, increased self-discipline requirements
	Opportunities	Enhanced accessibility for diverse learners, integration of multimedia resources, global collaboration, continuous improvement, scalability
	Threats	Technological barriers, privacy and security concerns, equity issues, overreliance on technology, potential for disengagement

## Discussion

The results revealed mixed views on e-learning in medical education by both teachers and students. The quality of e-learning systems was totally moderate from the perspective of medical students. In addition, teachers and representatives of students reported mixed results in the interviews and Our Qualitative data supported the findings of the quantitative section and provided a deeper understanding.

The results showed that the course structure, the student support system, and the infrastructure and technology domains were significantly higher than the mean value. However, the teaching-learning strategies subscale mean score was lower than the average level, indicating that there is room for improvement in this aspect of the educational environment. According to a number of research, the relationship between effectiveness and appropriate e-learning strategies is extremely important [24]. Wilcha’s research has shown that educational methods and learner interaction are critical in e-learning [25]. The students’ representative and experts’ views revealed that lack of student engagement was a prominent issue observed in the online classes, and the strategies employed failed to effectively captivate students, leading to a noticeable absence of interest and a sense of boredom. The absence of face-to-face interactions with peers and instructors could deprive students of the social aspect of traditional classrooms, which may impact their motivation, sense of belonging, and ability to form meaningful relationships with fellow students and teachers [26]. The experts also emphasized the use of blended learning in medical education and believed that it is one of the most effective ways that can replace purely virtual courses. The weaknesses of virtual education present an opportunity to explore and implement innovative approaches that are better suited for virtual education [27]. To enhance student engagement and reduce boredom associated with traditional lecture-based approaches, active learning strategies such as case studies, group discussions, and virtual collaborative projects can be implemented [28]. Additionally, effective use of technology, including interactive online platforms, virtual reality simulations, and gamification elements, can create more dynamic and engaging learning experiences for students [29]. Wu et al. found that the most effective e-learning designs include interactive learning activities, learner motivation and enthusiasm, the right presentation technologies, and learning in the social and personal context of the learner [30]. These findings suggest that the successful implementation of e-learning strategies requires a thoughtful approach, focusing on learner engagement and experience. Therefore, the success of e-learning systems depends on the educational strategies used and their combination with appropriate technology [31]. One reason these facilities were not available during the COVID-19 pandemic outbreak was that most professors converted their face-to face courses into virtual ones without appropriate technology-based strategies [24]. Applying appropriate learning strategies in virtual environments takes time and requires the necessary skills to gradually achieve this goal [25].

Furthermore, the study found that student support systems were one of the most influential factors in student satisfaction, with the university investing in student services and technology to facilitate online education. Student support systems include all services that facilitate the learning of a specific learner prior to, during, and after learning [26–27]. In this study, the students received training and online support to participate in classes, and other software essential to online education. They had easy access to educational portals, instructors, and digital libraries, and the university created a learning management system to manage the process of e-learning. Student support technicians developed social media and included students from all faculties, who could remotely assist students with problem-solving and guide them. The students’ perceptions toward e-learning systems are consistent with the findings of this study. According to a number of studies, the student support system is one of the most influential factors in student satisfaction [28]. One possible reason for the higher quality reported in the student support system domain is that the university places a high priority on developing the system across all faculties. Moreover, the university has invested in the development of student services that are provided online, via a remote system, and by phone. They had easy access to educational portals, instructors, and the digital library. The university paid special attention to the application of technology in the majority of its services, which is one reason why the highest quality has been reported [29]. The university also created a learning management system (LMS) to manage the process of e-learning through networks that let students talk to each other and interact with the content of e-courses. ^[17]^ In addition, it provided online training and support for students’ participation in LMS, virtual classes, and software essential to virtual education. Additionally, student support technicians developed social media and included students from all faculties. So that they could remotely assist students with problem-solving and guide them. Content, as a significant challenge.

The study also highlighted the importance of teacher training and support in facilitating successful e-learning experiences. Participants stressed the need for teachers to be adequately trained in e-learning technologies and pedagogies to ensure that they are equipped to deliver high-quality online instruction. Additionally, participants emphasized the need for ongoing technical support to help students and teachers overcome any issues that arise.

Moreover, from the students’ perspective, the course structure received the highest quality score. According to some experts, a clearly structured course helps students understand what to expect in the course and what is expected of them each week, reducing stress and allowing them to better manage their time and organization [31]. One of the main advantages of e-learning identified by the teachers and students was its flexibility and convenience, allowing them to learn and teach at their own pace and in their own time, access educational resources efficiently, and receive feedback whenever they need it. This reduces their stress and allows them to better manage their time and organization [32]. An appropriate course structure also provides students with a greater sense of clarity, focus, and direction in their studies and increases discoverability, which is essential for online learners [20]. The present study revealed that students were provided with comprehensive information about the educational calendar, course plan, and curriculum. Additionally, having a clearly structured course gives students the ability to plan ahead, which is beneficial for their academic and career goals [33].

Additionally, e-learning platforms provide access to a wealth of resources, such as recorded lectures, interactive modules, and online textbooks, which can support and enhance learning. The experts also noted several weaknesses of virtual education, including technical challenges, self-motivation and discipline. They also identified the lack of appropriate infrastructure and virtual teaching tools for teachers, as well as their unfamiliarity with the hardware and software required for teaching and producing suitable [34]. This research disclosed a significant relationship between age, gender, grade, and test score. The result revealed that female students had significantly higher mean evaluation scores. Additionally, the mean evaluation score declined significantly with age. Fiorina et al. demonstrated that student satisfaction with the quality of virtual education decreases significantly with age and study years [25]. In addition, it appears that higher-semester students with more traditional education experience received a lower score for virtual education in the current study. Additionally, Aljaraideh et al. discovered that women are more interested in e-learning than men [35]. The research reveals that younger students, particularly females, have a greater preference for technology-oriented methods. They desire to study in digital environments, participate in virtual groups, and express their opinions in virtual groups [36–37]. The difference between the views of students and teachers regarding infrastructure and software highlights the gap between students who belong to the digital age and digital natives, and teachers who are mostly digital immigrants. To bridge this gap, teachers and need relevant training to increase student achievement.

## Limitation and recommendation

This research was conducted in a single state only. It is suggested that future research be carried out over a longer time period and with a larger sample size in order to obtain a more precise evaluation of medical students.

## Conclusion

This mixed-method study provided insights into the experiences of faculty members and medical students with e-learning during the COVID-19 pandemic. The integration of quantitative and qualitative data provided a more comprehensive understanding of the phenomenon under study. In conclusion, virtual education has several strengths and weaknesses, and there is a need to explore innovative approaches to enhance student engagement and reduce boredom. The lack of appropriate infrastructure and virtual teaching tools for teachers and students, as well as their unfamiliarity with the hardware and software required for teaching and producing suitable content, is a significant challenge that needs to be addressed. The use of blended learning in medical education is considered effective, and the shift from teacher-centered to learner-centered teaching approaches is an opportunity to explore innovative teaching approaches.

## Data Availability

The data supporting this study’s findings are available from the corresponding author on request.
